# Construction of Complex Structures Containing Micro-Pits and Nano-Pits on the Surface of Titanium for Cytocompatibility Improvement

**DOI:** 10.3390/ma12172820

**Published:** 2019-09-02

**Authors:** Guisen Wang, Yi Wan, Zhanqiang Liu

**Affiliations:** 1 Key Laboratory of High Efficiency and Clean Manufacturing, School of Mechanical Engineering, Shandong University, Jinan 250061, China; 2 National Demonstration Center for Experimental Mechanical Engineering Education, Shandong University, Jinan 250061, China; 3 Department of Mechanical Engineering, Tsinghua University, Beijing 100084, China; 4 Beijing Key Lab of Precision/Ultra-precision Manufacturing Equipment and Control, Tsinghua University, Beijing 100084, China

**Keywords:** titanium, surface modification, micro/nanostructure, cell response, biocompatibility

## Abstract

The surface topography of medical implants plays an important role in the regulation of cellular responses. Microstructure and nanostructure surfaces have been proved to enhance cell spreading and proliferation with respect to smooth surfaces. In this study, we fabricated a new structure including micro-pits and nano-pits on the surface of titanium via sandblasting, acid etching and chemical oxidation to investigate the influence of composite structures on cell behavior. Meanwhile, the surface properties and corrosion resistance of treated samples were also tested. The micro/nanostructured titanium surface comprising of micro-pits and nano-pits presented enhanced roughness and hydrophilicity. In addition, the corrosion resistance of the titanium substrate with micro-pits and nano-pits was significantly improved compared to that of polished titanium. More importantly, the micro/nanostructured titanium surface proved a good interfacial environment to promote osteoblast functions such as cell adhesion and spreading. Taken together, these results showed that the construction of micro/nanostructure on the titanium surface is an effective modification strategy to improve osteoblast cell responses.

## 1. Introduction

Titanium (Ti) and its alloys are commonly used for manufacturing dental and orthopedic implants because of their excellent biocompatibility and mechanical properties [1,2,3]. However, implant failures still occur frequently in clinical applications due to the low bonding strength and bonding speed of implant and bone tissues [4,5]. It has been reported that surface specificities (e.g., topography, chemical composition, hydrophilicity, roughness) have an important influence on the interaction of cells or tissue and implants [5,6,7]. Therefore, a great number of surface modification techniques on implants, such as alkali-heat treatment, anodic oxidation, micro-arc oxidation, and ion implantation, have been proposed to stimulate desired biological responses for enhancing osseointegration [8,9,10,11,12]. 

Surface topography is one of the key factors in the manufacture of medical Ti implants, and affects the response of cells to implants [13,14]. It is generally accepted that the microstructured surface is beneficial to increasing bone-implant contact and anchorage for improving the bonding strength of interface [5,15]. Nevertheless, natural bone is a hierarchical structure containing macroscale, microscale, sub-micro scale, nanoscale, and sub-nanoscale organizations [16]. The influence of micro-topography on the osseointegration of the implant is unsatisfactory. Nano-topography is also important for regulating the interaction between the implant and bone tissues. Previous studies have shown that the nanostructured surface was conducive to promoting cell attachment, proliferation, and differentiation [9,17,18,19,20]. This is considered relevant to the interaction of nanostructure and cell membrane receptors. From a biomimetic perspective, the micro/nanostructured surface can mimic the structure of bone tissue and present great potential for enhancing cell responses. Moon et al. [21] demonstrated that the synergistic effects of micro- and nano-textural surface significantly improved cell attachment, proliferation, and bone regeneration. Therefore, the fabrication of micro/nanostructure on Ti surface has attracted much scholarly attention. 

In recent years, many methods for constructing micro/nanostructure on the Ti surface have been developed. For example, Huang et al. [22] constructed micro/nanostructures on Ti surfaces via micro-arc to construct micro-crater and hydrothermal treatment for 24 h to form nanoplates. Experimental results showed that the micro/nanostructured surface contributed to enhancing cell spreading, adhesion, and proliferation. Zhao et al. [23] produced micro/nanostructures by constructing titania nanotubes on micro-topographies via the methods of acid etching and anodic oxidation, which improved the multiple osteoblast functions such as cell proliferation and mineralization. Despite previous studies reporting some promising results, further research is still required on the preparation of micro/nanostructures on the surface of Ti, their surface properties, as well as their biocompatibility. This will be conducive to enriching the surface modification strategies of implants.

Sandblasting with large grit and acid etching have been widely used to modify the surfaces of Ti implant in recent years, and this is one of the most successfully commercialized surface modification methods. However, it is difficult to produce homogenous nanostructures on the surface of Ti using this method. In addition, the oxide layer on the surface of titanium is also relatively thin. Chemical oxidation, as a simple method, has been used for constructing nano-pits on Ti surface [24]. With this approach, the thickness of the dioxide layer on the Ti surface is significantly increased [24]. Meanwhile, nanostructures fabricated on the surface also promote the growth of osteoblasts. To our knowledge, the influence of the complex structures including micro-pits and nano-pits on osteoblasts has seldom been reported. Accordingly, in this study, complex structures were constructed on the Ti surface through a combination of sandblasting, acid etching, and chemical oxidation. Following this, the surface topography, hydrophilicity, and corrosion resistance of differently treated Ti samples were measured by scanning electron microscopy, contact angle goniometer, and electrochemical workstation, respectively. Furthermore, the influence of micro/nanostructures on cell responses was also investigated in order to evaluate the biocompatibility of Ti samples. The purpose of this study was mainly to evaluate the feasibility of constructing complex structures containing micro-pits and nano-pits using the novel pathway combined with sandblasting, acid etching and chemical oxidation.

## 2. Materials and Methods 

### 2.1. Samples Fabrication

**Figure 1:**
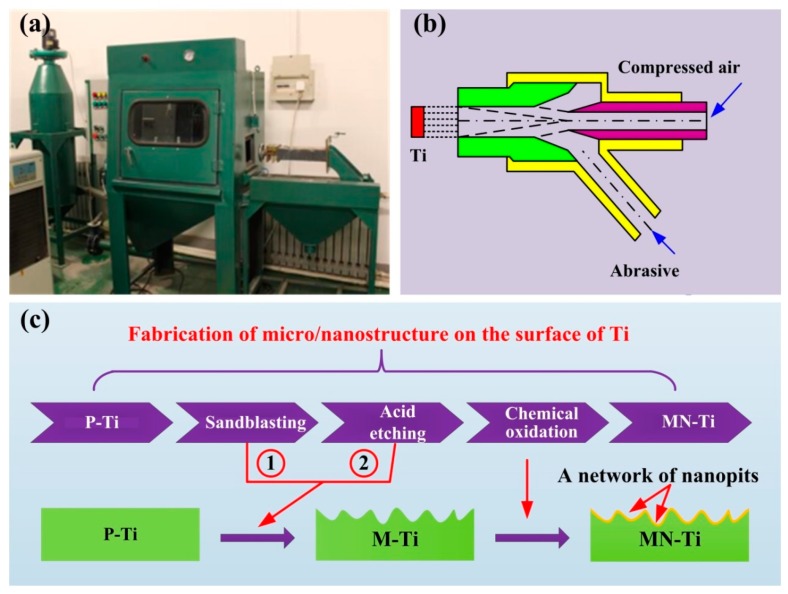
The equipment (**a**) and schematic diagrams (**b**) of the sandblasting process, and the process of fabrication of micro/nanostructure on the surface of Ti (**c**).

Pure Ti (TA2) was purchased from Baoji Titanium Industry (Shanxi, China), which was manufactured into a square block with a size of 10 × 10 × 1 mm^3^. The Ti substrates were polished with a polish-grinding machine and cleaned in acetone, anhydrous ethanol, and deionized water successively using an ultrasonically cleaner. The polished Ti was defined as P-Ti. After that, the samples were sandblasted with Al_2_O_3_ particles (125–150 µm) in a sandblasting device. The sandblasting was performed at a distance of 10 cm from the nozzle to the substrate in the vertical direction under a pressure of 0.34 MPa for 20 s. To remove the residual grit particles, the samples were immersed in a mixed solution of HF (2%) and HCl (1.8%) for 150 s. The treated samples were defined as M-Ti. Thereafter, the M-Ti samples were soaked in a piranha solution at 80 °C for 2 h to form nanostructure on the surface (MN-Ti). A schematic diagram of the fabrication process presented in Figure 1. 

### 2.2. Surface Characterization

Field-emission scanning electron microscopy (SEM, Carl Zeiss, Oberkochen, Germany) was used to evaluate the surface topography of different samples. The roughness and three-dimensional (3D) profile images were investigated by a laser scanning microscope (LSM, VK-X200K, Osaka, Japan). To evaluate the crystalline phase of treated samples, X-ray diffraction (XRD, D8 Advance, Karlsruhe, Germany) was used in this study. The scan was carried out in the 20–80° range with an angle of the incident beam of 0.02°. The water contact angle (CA) of the samples was measured by a contact angle goniometer (SL200KS, Boston, MA, USA). The distilled water used in this study was dropped onto the surface of samples by a syringe. The drop size of the distilled water was set as 2 μL. 

### 2.3. Corrosion Resistance Evaluation

The corrosion resistance of P-Ti, M-Ti, and MN-Ti samples was investigated by an electrochemical workstation (Corrtest, Wuhan, China). Physiological saline solution (0.9wt% NaCl, pH = 7) was used as the electrolyte solution [25]. The specimens (exposed area: 1 cm^2^) were immersed in the physiological saline solution for several minutes under open circuit potentials (OCP). Potentiodynamic polarization curves were measured according to our previous study [3].

### 2.4. Cell Culture

Mouse osteoblasts (MC3T3) were used to evaluate the biocompatibility of P-Ti, M-Ti, and MN-Ti samples in this study. MC3T3 cells were cultured in α-MEM that included 10% fetal bovine serum and 1% penicillin/streptomycin. The culture flask contained MC3T3 cells and culture medium were put in 37 °C incubators with the atmosphere of 5% CO_2_. The culture medium was refreshed every two days. Cell passage was carried out when MC3T3 cells fusion reached 70%-80%. Cells at passage 3–5 were seeded onto different sample surfaces to investigate their response to the sample surfaces. 

### 2.5. Cell Attachment 

The samples were removed from the culture medium and gently washed with PBS after 24 h of culture. Then, 4% of paraformaldehyde was used to fix the cells that grow on different samples. After that, the nucleus of osteoblasts was stained by Hoechst 33258 (Beyotime, Jiangsu, China) at room temperature. Finally, the number of cell nuclei was counted in three random fields on each sample by the confocal laser scanning microscope (CLSM, LSM 780, Oberkochen, Germany).

### 2.6. Cell Morphology

After 48 h of culture, MC3T3 cells cultured on the surfaces of P-Ti, M-Ti, and MN-Ti substrates were fixed for 30 min by paraformaldehyde at 4 °C and rinsed three times with PBS. Then, 0.2% Triton X-100 solution was used to permeabilize MC3T3 cells at 4 °C. After that, cells were stained with rhodamine-phalloidin (Beijing, China) and Hoechst 33258 (10 μg/mL) at room temperature according to the steps specified in the instructions successively. Finally, the strained cells on the surface of different samples were observed using CLSM.

### 2.7. Cell Proliferation

MC3T3 cells were seeded onto the different sample surfaces at a density of 2 × 10^4^ cells/well. MTT (Beijing, China) assay was used to investigate MC3T3 cells proliferation behavior at day 1, 4, and 7. At the end of each culture period, the samples were removed from the cell culture plate and washed with PBS. Then, the rinsed samples were transformed into a new plate that contained 1000 μL mixed solution (900 μL cell culture medium + 100 μL MTT solution) and placed in the incubator for 4 h at 37 °C. Next, the mixed solution was removed from the plate by pipette and 500 μL DMSO was added to each well. The absorbance of the solution (200 μL) was investigated by a spectrophotometric microplate reader at 490 nm.

### 2.8. Alkaline Phosphatase (ALP) Activity Assay

After culture for 7 days, MC3T3 cells adhered on P-Ti, M-Ti, and MN-Ti substrates were rinsed with PBS. Then, they were lysed by 0.1% Triton X-100. The total intracellular protein and ALP activity were investigated in accordance with the instructions. The ALP activity was normalized according to the method in Reference [26].

### 2.9. Mineralization Assay

Mineralization assay was carried out after culture for 21 days. MC3T3 cells adhered on Ti samples were stained with alizarin red according to the method in the literature [27]. Briefly, cells grown on different Ti surface were fixed with 4% paraformaldehyde and stained with 40 mM alizarin red. After that, 400 μL of acetic acid (10%, v/v) was added to each well with shaking. Subsequently, the cell monolayer was scraped from the Ti surface. They were transformed into new centrifuge tubes, respectively. The solution was heated to 85 °C for 10 min and centrifuged in a centrifuge. Next, the supernatant (200 μL) of each tube was removed, transferred into a new vial, and neutralized with ammonium hydroxide (200 μL, 10%, v/v). Finally, the absorbance of the reaction solution was investigated under a wavelength of 405 nm.

### 2.10. Statistical Analysis

The experimental data in this study were shown as mean ± standard deviation (SD). The statistical analysis was executed by GraphPad Prism 5 software with confidence levels of 0.95 and 0.99.

## 3. Results and Discussion

### 3.1. Surface Characterization

**Figure 2:**
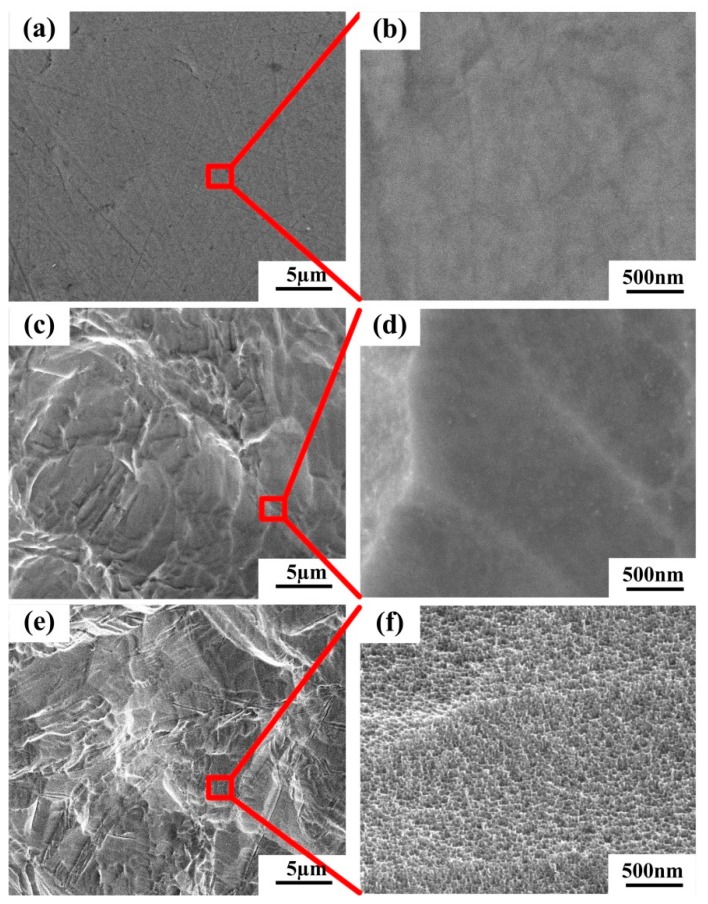
SEM images of (**a**,**b**) P-Ti, (**c**,**d**) M-Ti, and (**e**,**f**) MN-Ti.

**Figure 3:**
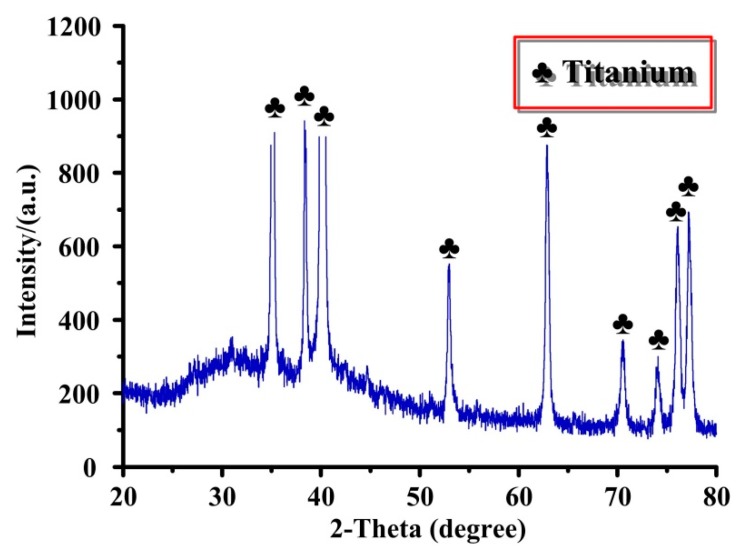
XRD spectrum of MN-Ti.

The surface topographies of different Ti substrates are shown in Figure 2. Apparently, the P-Ti substrate showed a smooth surface topography with visible scratches because of the influence of physical polishing (Figure 2a). After sandblasting and acid etching, many micro-pits appeared in the Ti substrate surface (Figure 2c). There was no obvious difference between M-Ti and MN-Ti substrates at low magnification (Figure 2c,e). However, a network with nano-pits presented on the surface of MN-Ti substrate (Figure 2f) due to the effect of chemical oxidation. To evaluate the physical structure of MN-Ti sample, we obtained its XRD spectrum. As shown in Figure 3, only a peak of Ti appeared on the MN-Ti surface, demonstrating the crystal phase was not altered after surface modification. It is generally accepted that sandblasting and acid etching can change surface topography, but not alter the crystal phase of the Ti surface [28,29]. In addition, the surface oxide layer on Ti substrate that treated with chemical oxidation is mainly composed of amorphous TiO_2_ [24]. Therefore, the XRD spectrum of MN-Ti only presented a peak of Ti. These results demonstrated that the micro/nanostructure containing micro-pits and nano-pits was successfully constructed on the surface of Ti by sandblasting, acid etching, as well as chemical oxidation. 

**Figure 4:**
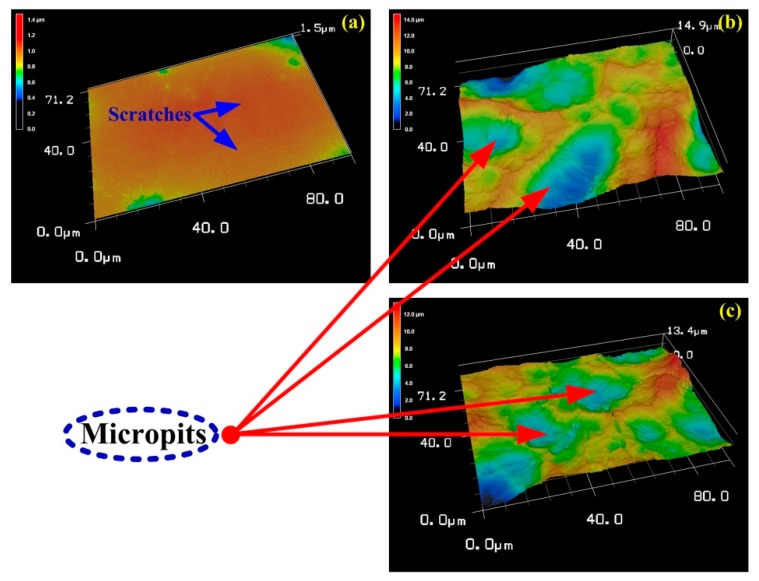
LSM images of different Ti samples: (**a**) P-Ti, (**b**) M-Ti, and (**c**) MN-Ti.

**Figure 5:**
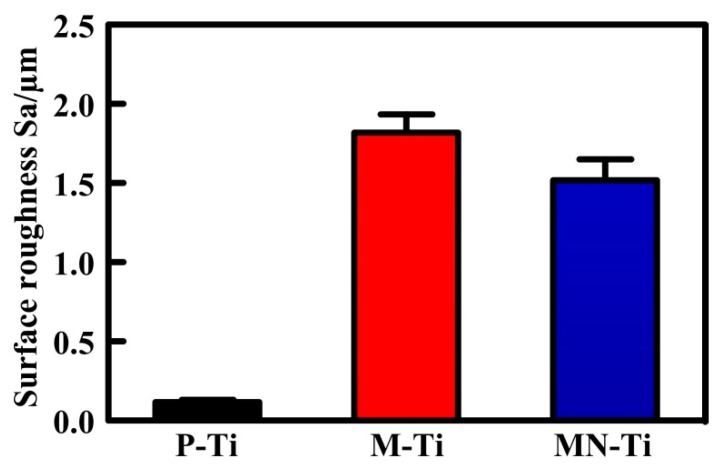
The surface roughness of P-Ti, M-Ti, and MN-Ti. Error bars represent means ± SD for n = 5.

The LSM images and surface roughness of P-Ti, M-Ti, and MN-Ti are displayed in Figure 4 and Figure 5, respectively. As shown in Figure 4, the P-Ti showed a flat surface with some scratches (Figure 4a), while the M-Ti (Figure 4b) and MN-Ti (Figure 4c) showed many micro-pits on their surfaces. No evident difference between M-Ti and MN-Ti was observed in the surface topography at low magnification. These results were in accord with the results of SEM. In Figure 5, it was clear that the roughness of M-Ti was rougher than that of P-Ti due to the existence of micro-pits. The roughness of MN-Ti was slightly decreased compared to M-Ti because the M-Ti samples were the further reaction in the piranha solution. In general, the surface roughness was good for the anchoring of implants to the bone and for promoting osteoblast differentiation [5,13,30,31]. Therefore, MN-Ti and M-Ti samples maybe exhibit better biocompatibility than P-Ti in this research.

### 3.2. Contact Angle Measurements

**Figure 6:**
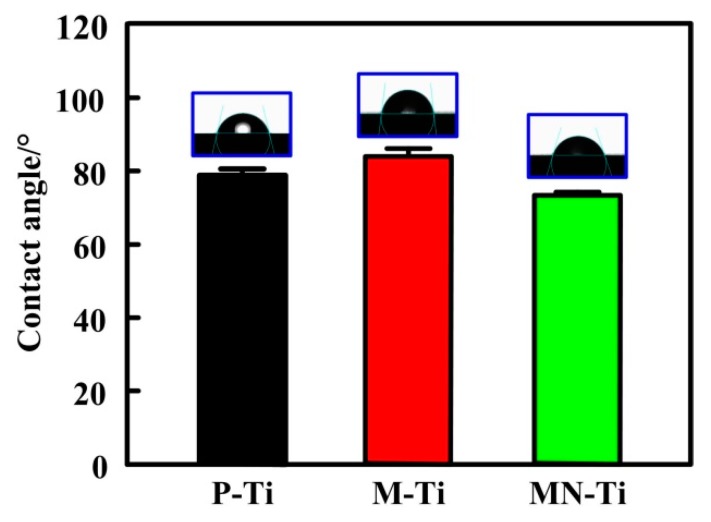
Water contact angles of P-Ti, M-Ti, and MN-Ti. Error bars represent means ± SD for n = 5.

The water contact angle was used to evaluate the effect of surface topography on the hydrophilicity of Ti samples. In biological systems, protein adsorption and cell adhesion are highly depended on the hydrophilicity of biomaterial surfaces [1]. As shown in Figure 6, the P-Ti substrate showed hydrophilic property and its water contact angle was 79.15°. This was due to the existence of hydrophilic hydroxyl group on the surface of polished Ti [5]. The contact angle of the M-Ti surface was 88.2°, implying that the hydrophilicity of Ti was reduced due to the effect of micro-pits. The contact angle of MN-Ti was the lowest among all groups. Therefore, the MN-Ti samples exhibited the best hydrophilicity, which may be more conducive to regulating cell responses than other samples.

### 3.3. Corrosion-Resistance Measurement

**Figure 7:**
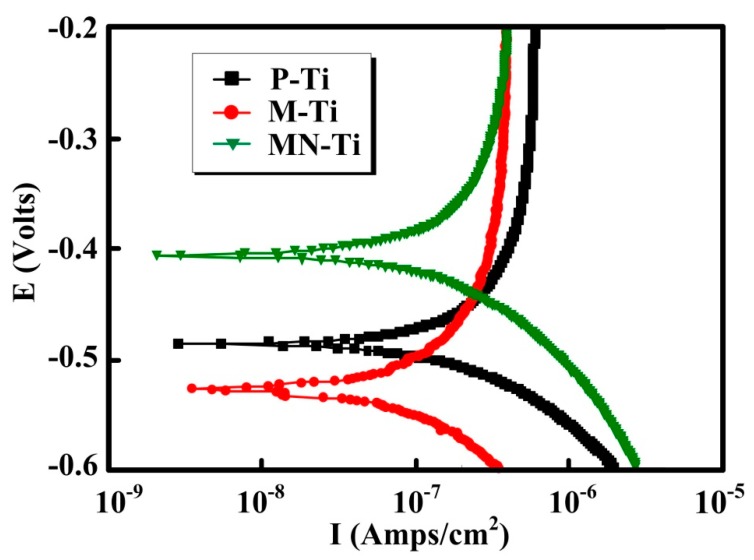
Polarization curves of P-Ti, M-Ti, and MN-Ti in NaCl solution.

For biomedical metallic materials such as Ti and Ti alloys, corrosion is unavoidable, which is responsible for implant failures. When the implant was implanted into the human body, biocorrosion, tribo-corrosion, and their combination would lead to the release of metal ions and metallic particles from the implant surface. This phenomenon would be more serious in the existence of fluoride ions. Therefore, biological complications (toxicity, carcinogenicity, and hypersensitivity) would happen due to the corrosion of biomaterials [32]. The polarization curves for P-Ti, M-Ti, and MN-Ti samples were measured in a NaCl solution, and the results were presented in Figure 7. The polarization curve shows the relationship between electrode potential and polarization current or polarization current density, which is widely used to evaluate the corrosion resistance of metallic materials. In general, the more electropositive of the polarization potential, the nobler the corroding material is [33]. The corrosion current represents the speed of corrosion of materials. The higher the corrosion current, the faster the corrosion rate. From Figure 7, it can be apparently seen that the corrosion potential of M-Ti was the lowest, and the corrosion potential was the highest. Thus, the corrosion resistance of M-Ti was the worst in all of the samples. This result indicated that the microstructure has a negative effect on the corrosion resistance of Ti in this study. It was found that the MN-Ti samples exhibited the highest corrosion potential and the lowest corrosion current. Hence, the corrosion resistance of the MN-Ti samples was the best in this study. It is demonstrated that chemical oxidation is helpful for improving the corrosion resistance of Ti. A recent study demonstrated that the corrosion resistance of Ti6Al4V is improved by turning in an oxygen-rich atmosphere, implying the increased thickness of the oxide film has a positive effect on the anticorrosion property [34]. It is reported that the thickness of the dioxide layer increased after chemical oxidation [24]. Therefore, the MN-Ti exhibited good corrosion resistance. 

### 3.4. Cell Attachment

**Figure 8:**
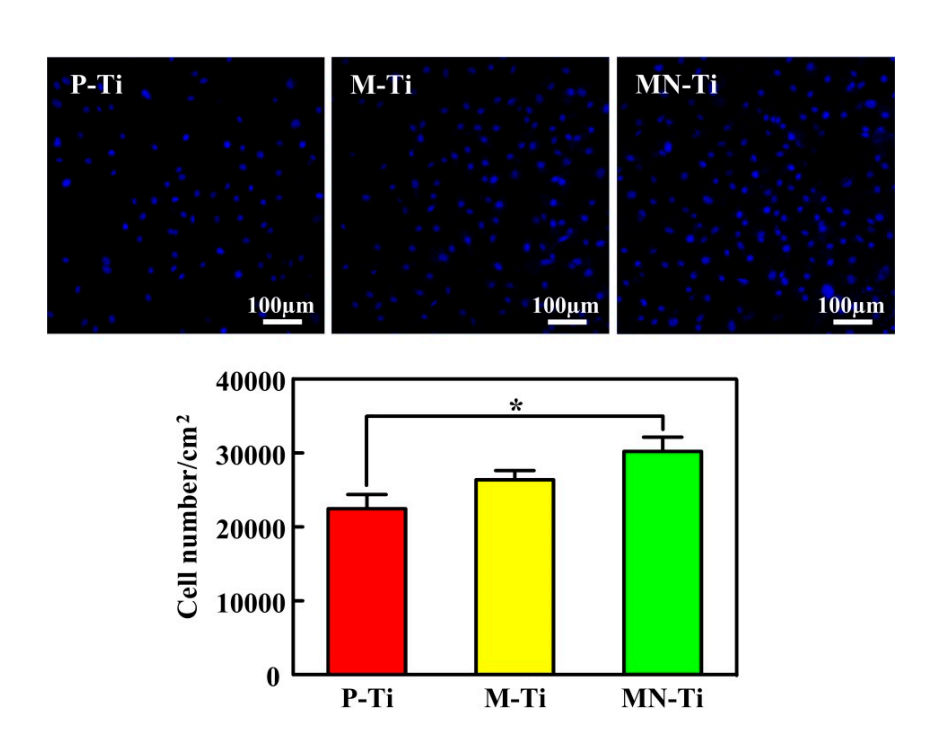
Initial adherent MC3T3 cell numbers measured by counting cells stained with Hoechst 33258 under a fluorescence microscope after 24 of incubation. * *p* < 0.05.

Cell attachment is the first event when cells come into contact with a material, which is an essential parameter for determining whether biomaterials are fit for medical use [9,35]. Therefore, excellent cell adhesion to the implant surface is needed for regulating subsequent cell behaviors and increasing the success rate of the implant [36,37,38]. The adherent cell number was measured by Hoechst 33258 staining, and the results were shown in Figure 8. The average cell numbers on the M-Ti and MN-Ti surfaces were higher than that on the P-Ti surfaces. However, there was no significant difference in the numbers of MC3T3 cells between P-Ti and M-Ti. The cell numbers cultured on the MN-Ti surfaces presented statistically larger than that on the P-Ti surfaces, which is in good accordance with previous work [28]. It is reported that microstructure can regulate cell microenvironment to enhance cell adhesion [39,40,41,42], the nanostructure can increase protein adsorption, regulate the formation of adhesion plaques, and change protein conformation to promote cell adhesion [43,44,45,46]. The MN-Ti samples that contained microstructure and nanostructure were most conducive to cell adhesion in our research. This is mainly because the micro-pits and nano-pits have a synergistic effect in improving the adhesion of osteoblasts. In addition, the excellent hydrophilicity of MN-Ti samples also helped for cell adhesion. 

### 3.5. Cell Morphology

**Figure 9:**
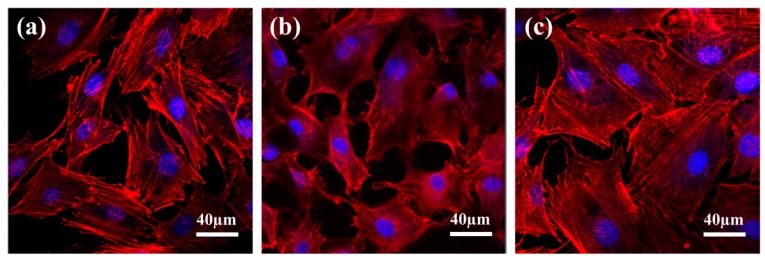
Fluorescent micrographs images of attachment of MC3T3 cells on P-Ti surface (**a**), M-Ti surface (**b**), and MN-Ti surface (**c**). Note: actin, red; nuclei, blue.

The morphology of MC3T3 cells adhered on different specimens was displayed in Figure 9. It was clear that the MC3T3 cells on samples P-Ti, M-Ti, and MN-Ti all exhibited spreading cell morphology. However, the MC3T3 cells possessed a larger spreading area on MN-Ti surfaces than those of other sample surfaces. Accordingly, MC3T3 cells grown on the MN-Ti surface exhibited a good stretch and strong adhesion ability [47]. Many previous studies showed that both microstructure and nanostructure can regulate cell spreading by affecting integrin signaling [46,48,49,50,51]. In this study, the MN-Ti substrates containing micro-pits and nano-pits were more propitious to cell spreading than other substrates. It is indicated that microstructure and nanostructure have a synergistic effect in improving cell spreading by affecting integrin signaling.

### 3.6. Cell Proliferation

**Figure 10:**
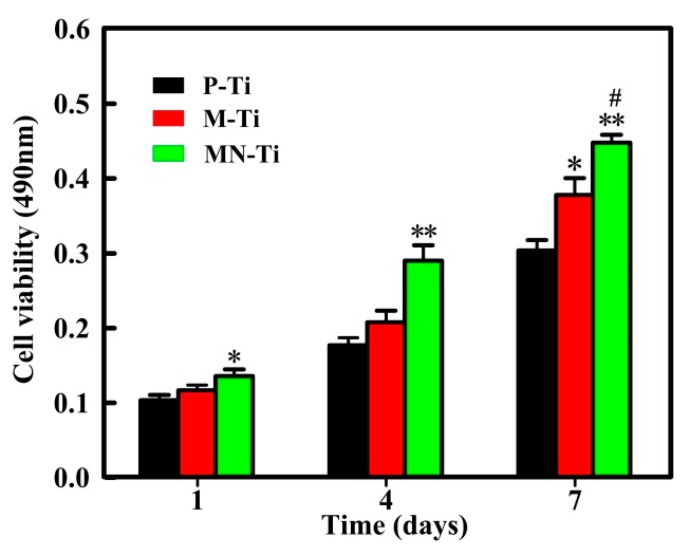
MTT assay of cell proliferation on different Ti samples after 1, 4 and 7 days of culture. ^#^ *p* < 0.05 compared with the M-Ti sample. * *p* < 0.05 and ** *p* < 0.01 compared with the P-Ti sample.

To evaluate the effect of different samples on the growth of MC3T3 cells, MTT assay was performed. Figure 10 presented the experimental results of cell proliferation. After 1 day of culture, cells grown on the MN-Ti surface were prominently higher than those on the P-Ti surface, which was consistent with the results of cell attachment. Although cells grown on M-Ti surface exhibited higher viabilities than those on the P-Ti surface, no significant differences existed between them at 1 and 4 days. The MC3T3 cells grown on the M-Ti and MN-Ti sample surfaces displayed remarkably higher cell viabilities than that on the P-Ti surfaces after culture for 7 days. Meanwhile, the viability of MC3T3 cells on MN-Ti surface was conspicuously higher than that on the M-Ti surfaces after 7 days of culture. The results proved that microstructure has a limited ability to regulate cell proliferation. Micro/nanostructure was more conducive to promoting cell proliferation than a single microstructure due to the existence of the nanostructure. It is most likely that the micro/nanostructure provides a wide range of dimensions from micron to nano for cell proliferation [52]. Moreover, the hydrophilic property of the micro/nanostructured surface also plays an essential role in promoting cell proliferation [53,54].

### 3.7. Cell Differentiation

**Figure 11:**
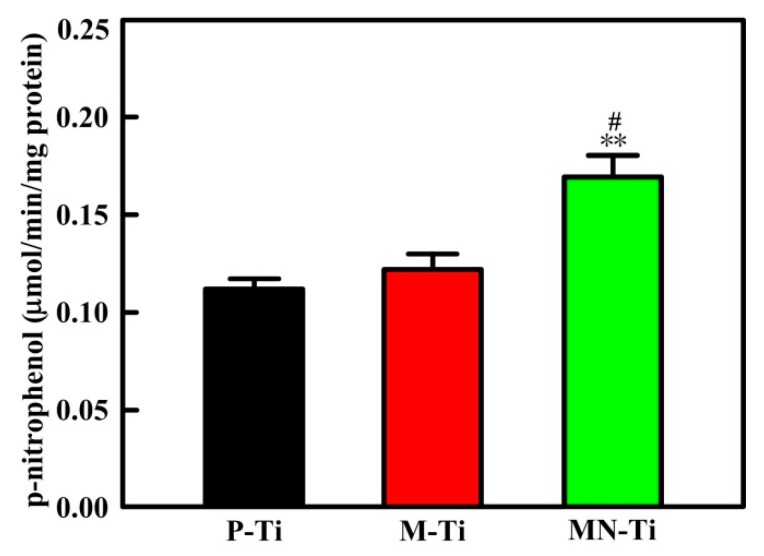
ALP activity of MC3T3 cells adhered to different Ti samples after culture for 7 days. ^#^ *p* < 0.05 compared with the M-Ti sample. ** *p* < 0.01 compared with the P-Ti sample.

In this study, ALP activity and mineralization were tested to assess the differentiation of MC3T3 cells grown on P-Ti, M-Ti, and MN-Ti surfaces. ALP is extensively used to evaluate cell differentiation at the early stage [9]. Mineralization is employed to investigate the level of cell differentiation [55]. The experimental results of ALP activity were presented in Figure 11. Osteoblasts grown on the MN-Ti surface showed significantly higher ALP activities than those on the P-Ti surface. However, the ALP activity of osteoblasts cultured on P-Ti and M-Ti showed no statistical difference. In addition, osteoblasts adhered onto the MN-Ti surface also presented higher ALP activities than those on the M-Ti surface. 

**Figure 12:**
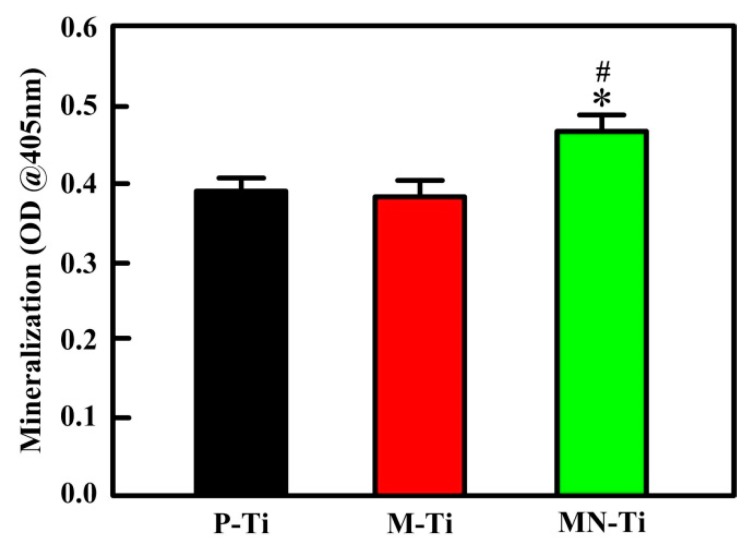
Mineralization of MC3T3 cells grown on different sample surfaces for 21 days. ^#^ *p* < 0.05 compared with the M-Ti sample. * *p* < 0.05 compared with the P-Ti sample.

Figure 12 presented the mineralization of MC3T3 cells cultured on P-Ti, M-Ti, and MN-Ti samples. Apparently, MC3T3 cells grown onto the MN-Ti substrate exhibited noteworthily higher mineralization than those of the P-Ti and M-Ti surfaces. There was no remarkable difference in cell mineralization products between P-Ti and M-Ti. These results demonstrated that the osteogenic differentiation of MC3T3 cells was improved on the MN-Ti surfaces due to the effect of microstructure and nanostructure. Wang et al. studied the mechanism of surface morphology on cell differentiation, they reported that micro/nanostructure can promote cell differentiation by regulating integrin-mediated signaling pathways (integrin-linked kinase/β-catenin pathway and Wnt/β-catenin pathway) [56,57]. 

## 4. Conclusions

In this study, micro/nanostructure consisting of micro-pits and nano-pits is fabricated on the surface of Ti by sandblasting, acid etching, and chemical oxidation. The roughness of the micro/nanostructured surface was significantly improved compared to polished Ti because of the influence of the micro-pits. There is no obvious difference in the surface roughness between M-Ti and MN-Ti. The micro-pits on the surface of Ti have a negative effect on corrosion resistance and hydrophilicity. However, the complex structures containing micro-pits and nano-pits displayed the enhanced hydrophilicity and corrosion resistance. The improvement of corrosion resistance is mainly due to the influence of chemical oxidation. Furthermore, MN-Ti sample has a great potential to improve the adhesion, spreading, proliferation, and differentiation of osteoblasts. This study provides a simple and effective method to construct the surface that contained micro-pits and nano-pits for improving the responses of osteoblasts to implants.
